# Abnormal vaginal bleeding in women of reproductive age: a descriptive study of initial management in general practice

**DOI:** 10.1186/1472-6874-8-7

**Published:** 2008-04-15

**Authors:** Corlien JH de Vries, Margreet Wieringa-de Waard, Cléo-Lotte AG Vervoort, Willem M Ankum, Patrick JE Bindels

**Affiliations:** 1 Division of Clinical Methods and Public Health, Department of General Practice, Academic Medical Center, University of Amsterdam, Meibergdreef 15, 1105 AZ Amsterdam, The Netherlands; 2 Department of Obstetrics and Gynaecology, Academic Medical Center, University of Amsterdam, Meibergdreef 15, 1105 AZ Amsterdam, The Netherlands

## Abstract

**Background:**

Abnormal vaginal bleeding (AVB) in women of reproductive age is a common reason for consulting a general practitioner. Nevertheless, how general practitioners (GPs) choose to initially manage AVB is largely unknown, as is the prevalence of underlying pathology of AVB in primary care.

**Methods:**

To investigate the initial diagnostic procedures and treatment for AVB used in general practice, we performed a descriptive study based on computerised medical records. New consultations for AVB in 2000 and 2001 were selected. Patient characteristics, diagnostic procedures and treatment were analysed.

**Results:**

In total, 270 new consultations were included. The majority of patients (75%) consulted the GP for AVB only once. GPs performed diagnostic procedures in 54% of all consultations. Overall, additional diagnostic procedures revealed abnormalities in 11% of women. However, the diagnostic procedures implemented by the GPs varied widely per bleeding type and contraceptive use. Anaemia was found in 36% of 45 women tested. Uterine fibroids were found in 41% of 27 women examined by ultrasound. Medication was prescribed in 34% of all consultations. A gynaecological referral was registered in 4% of all contacts.

**Conclusion:**

Initially, GPs tend to follow a policy of expectant management in women of reproductive age with AVB. However, when additional diagnostic procedures were performed, anaemia and uterine fibroids were found in a considerable number of women.

## Background

Abnormal vaginal bleeding (AVB) is a frequent reason for women of reproductive age to consult a general practitioner (GP). AVB can be categorised as excessive menstrual bleeding, irregular bleeding and intermenstrual including postcoital bleeding [1]. A population study carried out in Dutch general practices revealed an incidence of menorrhagia of 8 per 1000 women aged 25–44 years, per year, while in irregular bleeding, including intermenstrual and postcoital bleeding, the incidence is 17 per 1000 women aged 25–44 years, per year [2]. However, little is known about which diagnostic procedures are chosen by GPs to differentiate between causes of AVB and to implement its therapeutic management in general practice [3-5].

Since the late nineties, several national practice guidelines for the management of abnormal vaginal bleeding in general practice have been published [1,6,7]. In the Netherlands, the guideline 'Vaginal bleeding' from the Dutch College of General Practitioners, was published in 1992 and revised in 2001 [1]. Data on causes of AVB in primary care are scarce. Consequently, these guidelines were mostly based on data acquired in secondary care and gave no specific advice on diagnostic methods. To fill this gap, we studied the use and outcome of initial diagnostic procedures and the therapeutic management by GPs of women of reproductive age with AVB.

## Methods

This study was carried out in an urban primary healthcare centre which provides care to 11,500 patients, including 3,435 women at risk (20–55 years). The age and sex distribution of the practice population is largely comparable with the general Dutch population [8]. The centre submits data to the continuous morbidity registration network at the Department of General Practice at the Academic Medical Center, University of Amsterdam.

GPs working at the healthcare centre record all consultations on a computerised database. The medical problems presented during consultations are recorded using International Classification of Primary Care (ICPC) codes [9] and associated free text is used to report reason for consultation, diagnostic procedures performed, medication prescribed and referrals to secondary care. This information is then stored in an anonymous database which is available for analysis. From this database, we selected all recorded consultations concerning abnormal vaginal bleeding in women aged between 20 to 55 years between January 2000 and January 2002 using ICPC codes and truncated text words. The ICPC codes used were: menorrhagia (X06), irregular bleeding (X07), intermenstrual bleeding (X08), menopause (X11), bleeding after sexual intercourse (X13), endometritis/pelvic inflammatory disease (X74), malignancy of female reproductive organs (X77), uterine fibroids (X78), and injuries of female reproductive organs (X82). After a pilot test using the same database but a different time period, successful truncated text words were found to be: %blood loss%, %spotting%, %flow%, %contact bleeding%, %menstr%, %cycle%, %dysbalance%, %gyn%, %ovary%, %fibroid%, %menor%, %metror%. All recorded consultations in which the free text contained the truncated text words were also selected.

The text of all consultations identified by ICPC codes and text words, was read by two authors (CJHdV and CLAGV) and scored independently on the following issues: type of bleeding pattern, use of hormonal contraceptives, diagnostic procedures performed by GPs, any prescribed medication, and referrals to a gynaecologist. Any disagreement was resolved by consensus. Type of bleeding pattern was categorised according to the Dutch guideline on vaginal bleeding. The following three patterns were defined. Irregular bleeding i.e. non-cyclical bleeding, as a result of which menstrual bleeding can no longer be recognised. Excessive bleeding i.e. cyclical bleeding excessive in either volume or duration, and intermenstrual bleeding i.e. bleeding in the interval between recognisable periods [1]. The fourth category was a combination of bleeding patterns, irregular and intermenstrual bleeding or excessive and intermenstrual bleeding. No use of hormonal contraceptives, or the use of a copper intrauterine device were clustered and described as no hormonal contraceptive use. The use of oral contraceptive pills, depot medroxyprogesterone acetate or levonorgestrel-releasing intrauterine system was clustered and described as hormonal contraceptive use.

To assess initial management we selected only new consultations. A new consultation was defined as a consultation-free period of three months for AVB. As a consequence, consultations for AVB from January to April 2000 were not included, as it was not known whether there had been a consultation-free period of three months for AVB. The same holds for consultations without a three months follow-up, i.e. women who first presented between October 2001 and January 2002. Women who went for their first consultation in these months were excluded.

The number of consultations for the same type of bleeding was registered. A consultation after three months for a different type of bleeding was considered to be a new episode. These recurrent episodes were not analysed. Pregnant and postmenopausal women were excluded, as were those patients who had started hormonal contraception three months before consultation. The incidence of AVB is represented as the number of new episodes per 1000 women per year. The numerator concerns new episodes of AVB, the denominator concerns the population size of women at risk.

Management was described in terms of the number and proportion of patients undergoing physical examination (bimanual examination and speculum examination) or laboratory testing (cervical smear, Chlamydia test, pregnancy test, haemoglobin test), additional diagnostic procedures (ultrasonography), prescribed medication (hormonal, non-steroidal anti-inflammatory drugs, iron suppletion, other medical treatment) or referral to a gynaecologist. The categories of investigations were not mutually exclusive (i.e. a GP may have performed several diagnostic tests). Data were analysed in SPPS version 14.0.

## Results

### General characteristics

A total of 270 consultations by women with AVB were recorded and selected from the database. The incidence of new cases of AVB was 52 per 1,000 women per year. Most women (75%) consulted the GP only once for AVB. The median age of the women was 41 years (interquartile range 36–46). Overall reasons for consultation were mainly an irregular bleeding pattern (40%) and excessive bleeding (36%) (see Table 1). The majority of the women (70%) did not use hormonal contraceptives (Table 1). In this group excessive bleeding (43%) and irregular bleeding (42%) were almost equally often recorded as reason for consultation (see Table 2). Intermenstrual bleeding was the most frequently seen symptom (41%) in women using hormonal contraceptives (see Table 2). In three consultations, a combination of bleeding pattern was recorded (results not shown).

**Table 1:** General characteristics

**General characteristics**	n (%)*
Number of patients	270
Number of patients with one consultation only	203 (75)
Median age, yrs (interquartile range)	41 (36–46)
Type of bleeding	
Irregular	107 (40)
Excessive	98 (36)
Intermenstrual	62 (23)
Combination	3 (1)
HC use	
No **	188 (70)
Yes ***	82 (30)

Abbreviations: HC use: hormonal contraceptive use.* Numbers are n (%).** Included use of copper iud n = 14. *** Hormonal contraceptive pill: n = 65, depot medroxyprogesterone acetate or levonorgesterel-releasing intrauterine system: n = 17.

**Table 2:** Initial investigations by GPs in women with abnormal vaginal bleeding

	All types of bleeding	Irreg. HC-	Irreg. HC+	Excess. HC-	Excess. HC+	Interm. HC-	Interm. HC+
	n = 270*	**n = 78**	**n = 29**	**n = 80**	**n = 18**	**N = 28**	**n = 34**
History only	125 (46)	44 (56)	10 (35)	41(51)	8 (44)	7 (25)	14 (41)
Any investigation	145 (54)	34 (44)	19 (65)	39 (49)	10 (56)	21 (75)	20 (59)
Gyn. exam.	101 (37)	21 (27)	14 (48)	23 (29)	4 (22)	21 (75)	16 (47)
Cervical smear	33 (12)	2 (3)	6 (21)	4 (5)	0	10 (36)	10 (29)
Chlamydia test	26 (10)	3 (4)	3 (10)	1 (1)	0	11 (39)	7 (21)
Haemoglobin test	45 (17)	14(18)	3 (10)	21 (26)	5 (28)	1 (4)	1(3)
Pregnancy test	8 (3)	3 (4)	1 (3)	0	1(6)	3 (11)	0
Ultrasound	27 (10)	4 (5)	5 (17)	10 (13)	2 (11)	4 (14)	2 (6)

Abbreviations: Irreg.: irregular bleeding. Excess.: excessive bleeding. Interm.: intermenstrual bleeding. HC-: no hormonal contraceptive (included use of copper intrauterine device). HC+: hormonal contraceptive (hormonal contraception pill, depot medroxyprogesterone acetate of levonorgesterel-releasing intrauterine system). Gyn.exam: gynaecological examination (bimanual or speculum examination).

*Numbers are n (%).

### Diagnostic procedures

Overall, GPs used only history taking to assess bleeding symptoms in 46% of all consultations. However, the methods of assessment of AVB varied widely per type of bleeding and hormonal contraceptive use. In consultations concerning intermenstrual bleeding without hormonal contraceptive use, GPs used only the history as a diagnostic tool in 25% of consultations, in contrast to 56% in consultations concerning irregular bleeding without hormonal contraceptive use.

Physical examination (bimanual and/or speculum examination) was performed in 37% of all consultations, most frequently in consultations for intermenstrual bleeding without contraceptive use (21/28, 75%) (see Table 2). In cases of intermenstrual bleeding cervical smears (20/33) and testing for Chlamydia (18/26) were the most frequently carried out tests, whereas in consultations for excessive bleeding haemoglobin tests were most often performed (26/45). Ultrasound examination was used in 27 (10%) of all consultations, mainly in those women with excessive bleeding (12/27).

### Diagnostic test results

Overall, additional procedures revealed abnormalities in 11% of women. Findings on bimanual and speculum examination as noted by GPs were too diverse to interpret retrospectively as being either normal or abnormal. Of all 33 cervical smears performed, three were Pap stage 2 (borderline smear) [10]. One of the 26 Chlamydia tests was positive. A low haemoglobin level (< 7.5 mml/L) was noted in 16 of 45 tested women (36%), most frequently (14/26, 54%) in women with symptoms of excessive bleeding. None of the pregnancy tests were positive. Of the 27 ultrasound examinations, uterine fibroids were reported in 11 cases (41%). One was described as indistinctive, 13 (48%) were described as normal, while two results were missing (see Table 3). Uterine fibroids were found on 7/10 (70%) ultrasound examinations on women with excessive bleeding who were not taking hormonal contraceptives (results not shown).

**Table 3:** Abnormal findings in investigations

**Abnormal findings**	**n (n tests)**	**%**
Pap stage 2	3 (33)	9
Positive Chlamydia-test	1 (26)	4
Haemoglobin level < 7.5 mm/L	16 (45)*	36
Positive pregnancy test	0 (8)	0
Uterine fibroids on ultrasound	11 (27)**	41

* Three test results missing. ** Two test results missing.

### Initial management

Initially, in slightly less than two-thirds (62%) of all women, the GPs did not prescribe medication. Depending on bleeding pattern and hormonal contraceptive use, this percentage decreased to 43% (34/80) in excessive bleeding without hormonal contraceptive use and rose to 93% (26/28) in intermenstrual bleeding without hormonal contraceptive use. In about a quarter (65/270) of the consultations, hormonal treatment (hormonal contraceptive pill or progesterone) was prescribed. In 35% (28/80) of the consultations concerning excessive bleeding without hormonal contraception, hormonal treatment was prescribed. NSAIDs were prescribed in 5% of the consultations. Excessive bleeding was the main indication. In four percent of all cases, GPs referred the women to a gynaecologist (Table 4). Reasons for referral were findings on physical examination and blood testing (2/11), a history of gynaecological abnormalities (2/11), use of co-medication (1/11), specific diagnostic investigation or treatment not assessable by the GP (5/11) and on patient's request (1/11).

**Table 4:** Initial management by GPs in women with abnormal vaginal bleeding

	All types of bleeding	Irreg. HC-	Irreg. HC+	Excess. HC-	Excess. HC+	Interm. HC-	Interm. HC+
	n = 270*	**N = 78**	**n = 29**	**n = 80**	**n = 18**	**n = 28**	**n = 34**
No medication	167 (62)	48 (62)	21 (72)	34 (43)	9 (50)	26 (93)	28 (82)
Medication	92 (34)	25 (32)	8 (28)	45 (56)	8 (44)	2 (7)	2 (6)
Hormonal treatment	65 (24)	22 (28)	6 (21)	28 (35)	5 (28)	1 (4)	1 (3)
NSAID	13 (5)	2 (3)	1 (3)	8 (10)	2 (11)	0	0
Iron-suppletion	8 (3)	0	1(3)	7 (9)	1 (6)	0	0
Other	6 (2)	1 (1)	1 (3)	2(3)	0	1 (4)	1
Referral to gyn.	11 (4)	5 (6)	0	1 (1)	1 (6)	0	4 (12)

Abbreviations: Irreg.: irregular bleeding. Excess.: excessive bleeding. Interm.: intermenstrual bleeding. HC-: no hormonal contraceptive (included use of copper intrauterine device). HC+: hormonal contraceptive (hormonal contraception pill, depot medroxyprogesterone acetate of levonorgesterel-releasing intrauterine system). Referral to gyn: referral to gynaecologist.*Numbers are n (%).

## Discussion

In this study we found that GPs tend to initially follow a policy of expectative management in women of reproductive age who are seen for abnormal vaginal bleeding. The majority of the women (75%) consulted the GP only once. GPs tend to base their medical management on history taking only, and they were restrictive in their prescription of medication. However, GP's use of physical examination and additional diagnostic tests varied widely per bleeding type and pre-existing contraceptive use. If GPs performed additional diagnostic procedures, anaemia and uterine fibroids were the most frequently diagnosed conditions.

In this study, the symptoms and management of AVB were studied in a general practice setting. Because we used both truncated text words and ICPC codes, our study population was most likely complete and thus to have provided reliable information. The quality assurance programme in the network concentrated on detailed registration and data quality. Half-yearly meetings were organised, where feedback based on aggregated data were presented to practices, and differences were discussed. Since data can only be checked when registered in the medical records, it cannot be excluded that physical examinations were actually performed more often than was reported. Missing haemoglobin results and ultrasound findings could be explained by different pathways of performing these tests (at the healthcare centre or in a laboratory), or of reporting test results. The missing results are considered random and not related to the origin of AVB or outcome. The data were not collected recently, but since studies on symptoms of abnormal vaginal bleeding and its underlying pathology in primary care are still lacking, our data provide good insight in the management of AVB [11,12].

With two exceptions, the Dutch guidelines recommend a bimanual and speculum examination in all women, regardless of bleeding pattern. The two groups in whom it is not considered necessary to perform a physical examination are; virgins during the five years after the menarche and women with intermenstrual bleeding, who use hormonal contraceptives, do not have post-coital bleeding or are not at risk of sexually transmitted diseases [1]. The recently published NICE guideline 'Heavy Menstrual Bleeding' does not recommend a bimanual examination and/or speculum examination be initially carried out in women with heavy menstrual bleeding unless there are 'red flag' symptoms (postcoital bleeding, intermenstrual bleeding, pelvic pain or pressure symptoms) [13]. In our study bimanual and/or speculum examination was initially performed in 29% of women with excessive bleeding who did not use hormonal contraceptives, so the NICE guideline may reflect current practice more closely than the Dutch guideline [1,13]. However, we found that GPs seldom registered their reasons for ignoring the guideline's recommendations.

Variations in management found in the literature can be explained by different definitions of the observation period for AVB. For example in a study that analysed a general practice register, gynaecological examination was observed to be performed more frequently (42%) in women with excessive bleeding. The observation period in this study was substantially longer (four years) and episode-based [3].

Most of the investigations performed by the GPs in our study resulted in normal findings, especially in women with intermenstrual bleeding. In a 2006 review of postcoital bleeding and the risk of cervical cancer in primary care, Shapley states that symptoms are common and the presence of cancer is a rarity [11]. However, in our study we found a low haemoglobin level in 36%, and, on ultrasound, uterine fibroids in 41% of the patients examined. There is still little known about the prevalence of these abnormalities in primary care [1,12,13]. Other studies estimate the prevalence of uterine fibroids in patients referred with abnormal uterine bleeding to be between 30%–60% [14-16]. The causal relation between AVB and uterine fibroids is still under discussion. We raise the question of whether uterine fibroids are being under-diagnosed by GPs in women with symptoms of abnormal vaginal bleeding, because of the limited use of ultrasonography [17,18]. To answer this question the use of ultrasonography should be studied in a larger primary care population.

In the Netherlands hormonal treatment (oral contraceptive pill or progestagens) is the preferred medical treatment in all types of AVB with no underlying cause [1]. Our study found that hormonal treatment was mainly prescribed in cases of excessive bleeding (35% in consultations without hormonal contraceptive use). However, in slightly less than two-thirds of the consultations GPs did not prescribe any medication. This was higher than in an episode-based study in which 23% of the women received no medication [3]. We found that most women with AVB consulted their GP only once. It is questionable whether expectant policy with regard to medication prescription is wise, as studies have established that interference in daily life by heavy periods is an important factor for consulting a GP, and that women prefer treatment which improves their ability to manage menstruation [19,20]. However, as patients rarely returned to their GP with the same problem, the limited and mainly hormonal treatment options available in primary care may not be a preferable alternative, especially for patients aged 40 and older. The explanation and reassurance from the GP is presumably enough for the patient to accept a policy of expectant management. We think it unlikely that patients went elsewhere as in the Dutch health system, patients are registered with only one general practice and it is not possible to consult other GPs.

Appropriate medical treatment may prevent referral to a gynaecologist. Our study demonstrated that in four percent of all consultations women were initially referred to a gynaecologist. In another study, the mean annual referral rate to gynaecology for menorrhagia was nine per 1000 women, aged 30–49 years per year [3]. In our study it was initially one per 1000 women per year in this subgroup.

## Conclusion

In conclusion, this study shows that GPs appear to regard AVB as a problem that initially only needs limited further evaluation. However, depending on the type of AVB, there are good reasons for implementing a more active policy. Ultrasonography may be of use to allow GPs to establish whether uterine abnormalities are actually responsible for AVB.

## Competing interests

The author(s) declare that they have no competing interests.

## Authors' contributions

CJHdV participated in acquisition of data, analysis and interpretation of data and drafting of the manuscript. CLAGV participated in acquisition of data and analysis. MWdW participated in analysis and interpretation of data and drafting of the manuscript, critical revision of the manuscript, and supervision. PJEB and WMA participated in the design of the study, and critical revision of the manuscript. All authors have read and approved the final manuscript.

## Pre-publication history

The pre-publication history for this paper can be accessed here:


